# Editorial: Zebrafish as a model for pharmacological and toxicological research

**DOI:** 10.3389/fphar.2022.976970

**Published:** 2022-08-08

**Authors:** Carla Denise Bonan, Anna Maria Siebel

**Affiliations:** ^1^ Laboratório de Neuroquímica e Psicofarmacologia, Programa de Pós-Graduação em Biologia Celular e Molecular, Escola de Ciências da Saúde e da Vida, Pontifícia Universidade Católica do Rio Grande do Sul, Porto Alegre, RS, Brazil; ^2^ Instituto de Ciências Biológicas, Universidade Federal do Rio Grande-FURG, Rio Grande, Brazil

**Keywords:** drug discovery, toxicicity, behavior, development, high-throughput screening

Zebrafish (*Danio rerio*) is a small freshwater teleost widely used as an *in vivo* vertebrate model system for biomedical research. This species has a fully sequenced genome, high fecundity, external and rapid development, optical transparency during the developmental period, several physiological similarities with humans, and endophenotypes similar to human diseases (Kimmel et al., 1995; Howe et al., 2013; Kalueff et al., 2013). The research employing zebrafish embryos and larvae can provide massive knowledge about pharmacological and toxicological effects in high throughput drug screening (Petersen et al., 2022). In addition, the complex behavioral repertoire of adult zebrafish, its sensitivity to drugs, and the ability to respond to them similarly to humans support their utility for pharmacological and toxicological research (Rico et al., 2011; Zanandrea et al., 2020).

The use of pharmacological and toxicological approaches to different developmental stages of zebrafish is relevant to deepen the knowledge of drugs and toxicants’ mechanisms. Therefore, this Research Topic aimed to collect studies that exploit the zebrafish model as a tool for pharmacological and toxicological research. After the joint efforts of the journal, editors, reviewers, and contributors, a total of fourteen high-quality articles were published. So, we prepared a detailed summary for these fourteen articles as follows.

Alijevic et al. investigated the neuropharmacological *in vitro* and *in vivo* effects of three alkaloids—nicotine, cotinine, and anatabine. Natural nicotinic alkaloids induced an anxiolytic-like behavior, and this effect depends on the activation of nAChRs and regulation of other neurotransmitter systems, such as noradrenergic and dopaminergic systems. Organic cation transporters (OCTs) facilitate the transport of cations and other compounds between extracellular fluids and cells. Gould et al. characterized *in vivo* uptake to the brain and the high-affinity brain membrane binding of the mammalian OCT blocker 1-1′-diethyl-2,2′cyanine iodide (decynium-22) in zebrafish. Obtained data showed that D-22 can reach the zebrafish brain and induce anxiolytic effects, decreasing anti-predator dorsal camouflaging. Monoamine oxidases (MAO) catalyze the oxidative deamination of a variety of monoamines, promoting a critical role in neuromodulation. Jaka et al. demonstrated the anxiolytic-like effects of natural MAO inhibitors on novel environment-induced anxiety in zebrafish. Vossen et al. showed for the first time that male zebrafish showed more severe behavioral impairments than females when exposed to ethanol, showing the importance of clearly including sex and time course as factors in behavioral experiments with adult zebrafish.

Ciclosporin A is a powerful immunosuppressant widely used in clinics. Wan et al. observed that ciclosporin A promoted cardiac toxicity in zebrafish larvae, which may be related to an up-regulation of Wnt signaling and oxidative stress.

Pesticides contaminate aquatic systems, which may impact non-target organisms such as fish. Zaluski et al. demonstrated changes in survival, hatching, and morphological parameters after acute exposure to atrazine and diuron commercial formulations in zebrafish embryos and larvae. These findings highlight the relevance of additional studies on the sublethal effects of these compounds, as well as the comparison of commercial formulas vs. isolated active ingredients. Chackal et al. demonstrated that nanoplastics and the Flame Retardant BDE-47, detected in the aquatic environment, promote cumulative metabolic disruption in zebrafish larvae and that co-exposure exacerbates this effect. These data raised concerns about the influence of these compounds in aquatic systems and on human health.

Related to the visual system, nerve growth factor (NGF) is a neurotrophin with an important role in ocular homeostasis. Cocchiaro et al. investigated the expression of NGF receptors in adult zebrafish retina and showed that intravitreal (IV) administration of rhNGF can increase zebrafish retinal regeneration in this model. In a review article, Cohen et al. discussed the role of estrogenic and thyroidogenic signaling in the modulation of the development and function of the visual system.

Regarding neurological disorders, Milder et al. developed a method to achieve a more robust screening of anti-seizure drugs using a seizure model in zebrafish. The findings can help us connect physiological and behavioral responses to anti-seizure drugs and better assess anti-seizure drug efficacy. Doyle and Croll discussed the Parkinson’s disease models developed in zebrafish and the benefits and advantages of this species in comparison to other animal models.

Kim et al. performed a screen of 1,403 bioactive small molecule compounds using transgenic larval zebrafish, characterized by dopamine neuron loss. The study showed, by the combination of *in vivo* imaging-based screen and bioinformatic analysis, an approach for identifying hit-to-lead candidates and unknown pathways and targets involved in dopamine neuron protection. Bedrossiantz et al. demonstrate that zebrafish, as a poikilothermic animal, is a suitable model to investigate binge-like methamphetamine neurotoxicity without hyperthermia as a confounding effect.

Finally, Winter et al. tested a combination of *in silico* analysis of clinical data with *in vivo* assessment of CRISPR/Cas9-mediated mutation in zebrafish crispant. The study showed evidence of the role of three genes in cardiovascular development and function, as well as the potential effect of loss of gene function on organ system pathophysiology.

In conclusion, this Research Topic has provided new experimental data and updated reviews. Wonderfully, this Research Topic advanced the understanding and updated insights on zebrafish as a model for translational pharmacology and toxicology.

## References

[B1] Howe K. Clark M. D. Torroja C. F. Torrance J. Berthelot C. Muffato M. (2013). The zebrafish reference genome sequence and its relationship to the human genome. Nature 496 (7446), 498–503. Erratum in: Nature. 2014 Jan 9;505(7482):248. 10.1038/nature12111 23594743 PMC3703927

[B2] Kalueff A. V. Gebhardt M. Stewart A. M. Cachat J. M. Brimmer M. Chawla J. S. (2013). Towards a comprehensive catalog of zebrafish behavior 1.0 and beyond. Zebrafish 10 (1), 70–86. 10.1089/zeb.2012.0861 23590400 PMC3629777

[B3] Kimmel C. B. Ballard W. W. Kimmel S. R. Ullmann B. Schilling T. F. (1995). Stages of embryonic development of the zebrafish. Dev. Dyn. 203 (3), 253–310. 10.1002/aja.1002030302 8589427

[B4] Petersen B. D. Bertoncello K. T. Bonan C. D. (2022). Standardizing zebrafish behavioral paradigms across life stages: an effort towards translational pharmacology. Front. Pharmacol. 13, 833227. 10.3389/fphar.2022.833227 35126165 PMC8810815

[B5] Rico E. P. Rosemberg D. B. Seibt K. J. Capiotti K. M. Da Silva R. S. Bonan C. D. (2011). Zebrafish neurotransmitter systems as potential pharmacological and toxicological targets. Neurotoxicol. Teratol. 33 (6), 608–617. 10.1016/j.ntt.2011.07.007 21907791

[B6] Zanandrea R. Bonan C. D. Campos M. M. (2020). Zebrafish as a model for inflammation and drug discovery. Drug Discov. Today 25 (12), 2201–2211. 10.1016/j.drudis.2020.09.036 33035664

